# Hypoxia-Inducible Factor Activation Protects the Kidney from Gentamicin-Induced Acute Injury

**DOI:** 10.1371/journal.pone.0048952

**Published:** 2012-11-08

**Authors:** Jeong-myung Ahn, Sun Jin You, Yun-Mi Lee, Se-Won Oh, Shin-young Ahn, Sejoong Kim, Ho Jun Chin, Dong-Wan Chae, Ki Young Na

**Affiliations:** 1 Department of Internal Medicine, Maryknoll Hospital, Busan, Korea; 2 Department of Internal Medicine, Seoul National University Bundang Hospital, Seongnam, Korea; 3 Department of Internal Medicine, Eulji General Hospital, Seongnam, Korea; 4 Seoul National University College of Medicine, Seoul, Korea; National Cancer Institute, United States of America

## Abstract

Gentamicin nephrotoxicity is one of the most common causes of acute kidney injury (AKI). Hypoxia-inducible factor (HIF) is effective in protecting the kidney from ischemic and toxic injury. Increased expression of HIF-1α mRNA has been reported in rats with gentamicin-induced renal injury. We hypothesizd that we could study the role of HIF in gentamicin-induced AKI by modulating HIF activity. In this study, we investigated whether HIF activation had protective effects on gentamicin-induced renal tubule cell injury. Gentamicin-induced AKI was established in male Sprague-Dawley rats. Cobalt was continuously infused into the rats to activate HIF. HK-2 cells were pre-treated with cobalt or dimethyloxalylglycine (DMOG) to activate HIF and were then exposed to gentamicin. Cobalt or DMOG significantly increased HIF-1α expression in rat kidneys and HK-2 cells. In HK-2 cells, HIF inhibited gentamicin-induced reactive oxygen species (ROS) formation. HIF also protected these cells from apoptosis by reducing caspase-3 activity and the amount of cleaved caspase-3, and -9 proteins. Increased expression of HIF-1α reduced the number of gentamicin-induced apoptotic cells in rat kidneys and HK-2 cells. HIF activation improved the creatinine clearance and proteinuria in gentamicin-induced AKI. HIF activation also ameliorated the extent of histologic injury and reduced macrophage infiltration into the tubulointerstitium. In gentamicin-induced AKI, the activation of HIF by cobalt or DMOG attenuated renal dysfunction, proteinuria, and structural damage through a reduction of oxidative stress, inflammation, and apoptosis in renal tubular epithelial cells.

## Introduction

Gentamicin is an aminoglycoside antibiotic that is widely used in the treatment of gram-negative bacterial infections. However, its clinical use has been limited because gentamicin-induced acute kidney injury (AKI) occurs in approximately 20% of patients [1]. An important cytotoxic mechanism of gentamicin is the induction of apoptosis, which has been reported in renal proximal tubule cells and mesangial cells [2], [3]. The mitochondrial pathway is known to be involved in gentamicin-induced apoptosis [4], [5], and reactive oxygen species (ROS) have been suggested to be important mediators of this mitochondria-mediated apoptosis [5], [6].

Hypoxia-inducible factor (HIF), a heterodimeric transcription factor composed of an oxygen-sensitive α subunit and a constitutively expressed β subunit, is an important regulatory factor in the defense mechanisms against hypoxia [7]. In normoxia, the α subunit rapidly undergoes oxygen-dependent hydroxylation of proline residues by prolyl hydroxylases (PHDs). The hydroxylated α subunit recruits the von Hippel-Lindau protein, which in turn tags the α subunit with ubiquitin groups and targets it for degradation within the proteasome [8], [9], [10]. In hypoxia, the α subunit escapes prolyl hydroxylation and binds to the β subunit, and the heterodimeric HIF translocates to the nucleus and activates the transcription of genes involved in erythropoiesis, angiogenesis, cell metabolism, cell growth, and apoptosis [11]. Therefore, PHD inhibitors stabilize HIF by inhibiting its degradation, thus enhancing HIF activity. Cobalt and dimethyloxalylglycine (DMOG) have been known to inhibit PHDs and activate HIF [12].

The up-regulation of HIF in various kidney disease models, including ischemic or nephrotoxic acute kidney injury and chronic kidney disease [13], [14], [15], [16] suggests that HIF plays an important role in protection against kidney injury. Recently, we reported that HIF activation protected against podocyte injury in a rat remnant kidney model with a reduction of oxidative stress [17]. The antioxidant effect of HIF has also been reported in renal ischemia-reperfusion injury and diabetic nephropathy [18], [19]. Moreover, HIF activation reduced renal tubular cell apoptosis in cisplatin-induced nephrotoxicity [14]. Dose- and time-dependent elevation of renal HIF-1α mRNA levels was reported in animal studies of gentamicin-induced nephrotoxicity [20]. Therefore, it is postulated that HIF may be involved in gentamicin-induced renal injury. Due to the interest in studying the role of HIF in gentamicin-induced AKI through modulation of HIF activity, we investigated, *in vitro* and *in vivo,* whether HIF has protective effects on renal tubule cell injuries induced by gentamicin, and we investigated the mechanism of such an effect.

## Materials and Methods

### Ethics Statement

All animal procedures were approved by Institutional Animal Care and Use Committee of Medical Science Research Institute, Seoul National University Bundang Hospital (BA 0810-033/037-01).

### Cell Culture

We cultured HK-2 cells (ATCC CRL-2190), which are proximal tubular epithelial cells derived from normal human kidney tissue, using Renal Epithelial Basal Medium (Lonza Walkersville Inc., Walkersville, MD, USA) with the recommended supplements included in the REGM Singlequot Bulletkit. The cells were fed two to three times weekly and subcultivated via trypsinization when near confluence. HK-2 cells between passages 10 and 25 were used for these experiments.

### Cell Treatment

The cells were divided into four groups; 1) control cells, 2) gentamicin-treated cells, 3) gentamicin-treated cells with cobalt pre-treatment, and 4) gentamicin-treated cells with DMOG pre-treatment. Before the experiments, the cells were incubated in basal medium in the absence of supplements for 24 h. Gentamicin (Sigma, St. Louis, MO, USA) and cobalt (Sigma, St. Louis, MO, USA) were dissolved in 0.9% saline solution. DMOG (Cayman, Ann Harbor, MI, USA) was dissolved in dimethyl sulfoxide (DMSO; Sigma, St. Louis, MO, USA) solution. On the day of the experiment, the cells were pre-treated with 150 µM of cobalt or 1 mM of DMOG for 24 h to activate HIF-1α. Previous studies demonstrated that no adverse effects were observed at these doses of cobalt or DMOG in the cells [21], [22]. The cells were then treated with 3 mM of gentamicin for another 24 h.

### Detection of Apoptosis

Gentamicin-mediated apoptosis in HK-2 cells was detected by enzymatically labeling DNA strand breaks using TUNEL staining. TUNEL staining was performed with a Cell Death Detection kit (Roche, Mannheim, Germany). To reveal the total nuclei, the same slides were stained with 4′,6′-diamidino-2-phenyindole (DAPI) in phosphate-buffered saline. Cells with apoptotic nuclei were counted in at least 10 different fields and were expressed as the percentage of the total cells counted. Additionally, TUNEL staining was performed on the kidney tissue of experimental animals using the same kit. Cells with apoptotic nuclei were counted in more than 20 consecutive fields under×200 magnification.

A caspase-3 activity assay was performed using of the Caspase-3/CPP32 Fluorometric Assay Kit (BioVision, Mountain View, CO, USA). The cells were incubated in cell lysis buffer and centrifuged at 14,000 rpm, and the supernatants were incubated with DEVD-AFC (specific substrate for caspase-3) at 37°C for 1 h. The activity was assayed using a fluorometer.

### Detection of Intracellular ROS

Oxidation-sensitive DCFH-DA dye (Life Technologies, Carlsbad, CA, USA) was used to determine intracellular production of ROS. The cells were loaded with DCFH-DA at a final concentration of 10 µM and were incubated at 37°C for 30 min. Next, the cells were washed with phosphate-buffered saline, removed from the dishes by scraping, and measured for fluorescence intensity. Fluorescence was measured with a fluorescence spectrophotometer at 490 nm of excitation and 526 nm of emission.

**Table 1:** Renal parameters of experimental animals.

	Control	Gentamicin	
	Vehicle (n = 5)	Vehicle (n = 6)	Cobalt (n = 6)
BUN (mg/dL)	19.2±2.5	27.3±13.8	17.8±2.4
Cr (mg/dL)	0.52±0.03	0.74±0.2*	0.64±0.07*
Cl_cr_ (ml/min/100 g)	0.41±0.06	0.27±0.07*	0.52±0.28* #
Urine PCR	0.44±0.09	3.27±1.73*	1.51±0.51* #

* P<0.05 vs. control group;

# P<0.05 vs. gentamicin-treated group;

Cr, creatinine;

Cl_Cr_, creatinine clearance normalized per body weight; PCR, protein-to-creatinine ratio.

### Western Blotting Analysis of Cellular Proteins

HK-2 cell lysate proteins were applied to each lane and analyzed by Western blot. Antibodies to HIF-1α (BD Bioscience, Franklin Lakes, NJ, USA), β-actin (Santa Cruz Biotech, Santa Cruz, CA, USA), caspase-3 (Cell Signaling Technology, Danvers, MA, USA), and caspase-9 (BD Bioscience, Franklin Lakes, NJ, USA) were used for assay. Incubation with peroxidase-conjugated anti-rabbit or anti-goat IgG secondary antibodies (Pierce, Rockford, IL, USA) was followed by band visualization using enhanced chemiluminescence (ECL™ RPN 2106; Amersham Pharmacia Biotech, Buckinghamshire, UK). The band densities were quantified by densitometry (GS-700 Imaging Densitometry, Bio-Rad, Hercules, CA, USA).

### Animal Experiments

Male Sprague-Dawley rats (Orient Bio Inc., Seongnam, South Korea), weighing approximately 220 g, were used. The rats (*n* = 17) were randomly assigned to three groups: the control, gentamicin + vehicle (GM + VEH), and gentamicin + cobalt (GM + Co) groups. All of the rats were anesthetized with enflurane (Choongwae Pharma Corp., Seoul, South Korea), and osmotic minipumps (model 2ML1; Alzet, Palo Alto, CA, USA) were subcutaneously implanted to deliver 10 mg/kg/day of cobalt (GM + Co group) or vehicle (control and GM + VEH group) for 7 days. This dose of cobalt was confirmed not to be shown any adverse effects on the rats [22], [23]. The vehicle consisted of 0.9% saline. To induce AKI by gentamicin, 80 mg/kg of gentamicin (GM + VEH and GM + Co group) or 0.9% saline (control group) was injected intramuscularly once per day for 7 days. After 7 days of experimentation, the animals were anesthetized with ketamine and xylazine, blood samples were obtained, and the kidneys were collected. The left kidney from each rat was fixed in 10% phosphate-buffered formalin for morphologic and immunohistochemistry analyses. The right kidney was snap-frozen in liquid nitrogen and stored at −80°C for RNA and protein extraction.

### Physiologic Measurements

At the end of the experiments, the rats were weighed and placed in metabolic cages, and their urine was collected for 24 h. Urine volumes were measured, and protein concentrations were determined by spectrophotometric assay (as modified by Lowry using bicinchoninic acid reagent [Pierce, Rockford, IL, USA]). The BUN and creatinine levels in the serum and urine were measured using an automatic analyzer (ADVIA 1650, Siemens). The creatinine clearance was calculated using a standard formula and was adjusted for body weight.

### Renal Histologic and Immunohistochemical Analyses

Tissue for light microscopy and immunoperoxidase staining was fixed in formalin and embedded in paraffin. Three-micrometer sections were stained with hematoxylin and eosin (H&E). Indirect immunoperoxidase staining with anti-ED-1 antibody (Serotec, Oxford, UK) was performed, as described previously [17].

### Quantification of Morphologic Data

All analyses were performed in a blind manner. The sections were examined and assigned morphological injury scores, as reported by Houghton *et al*. [24]: 0 = normal; 1 = areas of focal granulovacuolar epithelial cell degeneration and granular debris in the tubular lumens, with or without evidence of tubular epithelial cell desquamation in small foci (1% of total tubule population involved in desquamation); 2 = tubular epithelial necrosis and desquamation easily seen but involving less than half of the cortical tubules; 3 = more than half of the proximal tubules showing desquamation and necrosis, and involved tubules are easily found; and 4 = complete or almost complete tubular necrosis. More than 10 consecutive fields were examined under×200 magnification and the results were averaged. The mean numbers of infiltrating macrophages (ED-1 positive cells) were calculated by averaging the total numbers of positive cells in more than 20 consecutive fields at×200 magnification.

### Semiquantitative Immunoblotting

Tissue preparation and semiquantitative immunoblotting were performed as described previously [17]. Briefly, whole kidneys were homogenized, and nuclear extracts were prepared from the homogenates. Protein concentrations were measured using a bicinchoninic acid protein assay kit (Sigma, St. Louis, MO, USA). Initially, “loading gels” were done on each sample set. The proteins from each sample were loaded into an individual lane, and electrophoresed on SDS-polyacrylamide mini-gels and then stained with Coomassie blue. Selected bands from these gels were scanned to determine the density and relative amounts of protein loaded in each lane. Finally, protein concentrations were “corrected” to reflect these measurements. The proteins were transferred to nitrocellulose membranes by electroelution. Western blots were analyzed with anti-HIF-1α antibodies.

### Real-time PCR

Real-time PCR was performed to evaluate the mRNA expression of vascular endothelial growth factor (VEGF) in the kidneys. Real-time PCR was performed using the Applied Biosystems 7500 Fast Real-Time PCR System (Applied Biosystems, Foster City, CA, USA), as described previously [17]. The primer/probe mixture was designed and provided by Applied Biosystems. VEGF mRNA and 18S rRNA were amplified in a 25 µL reaction volume with 10 µL of 2x TaqMan Universal Master Mix (Applied Biosystems) and 1 µL of –20× Assays-on-Demand (Rn00582935_m1 for VEGF and Hs99999901_s1 for 18S rRNA). Each sample was measured in triplicate. 18S rRNA was used for normalization. The mRNA expressions are presented as the relative values obtained from the rats in the control group.

### Statistical Analysis

All of the data are presented as means±s.d. The statistical analysis was performed using the Mann-Whitney U-test and the Kruskal-Wallis test with SPSS software (version 15.0. for Windows; SPSS Inc., Chicago, IL, USA). Statistical significance was indicated by *P*<0.05.

## Results

**Figure 1:**
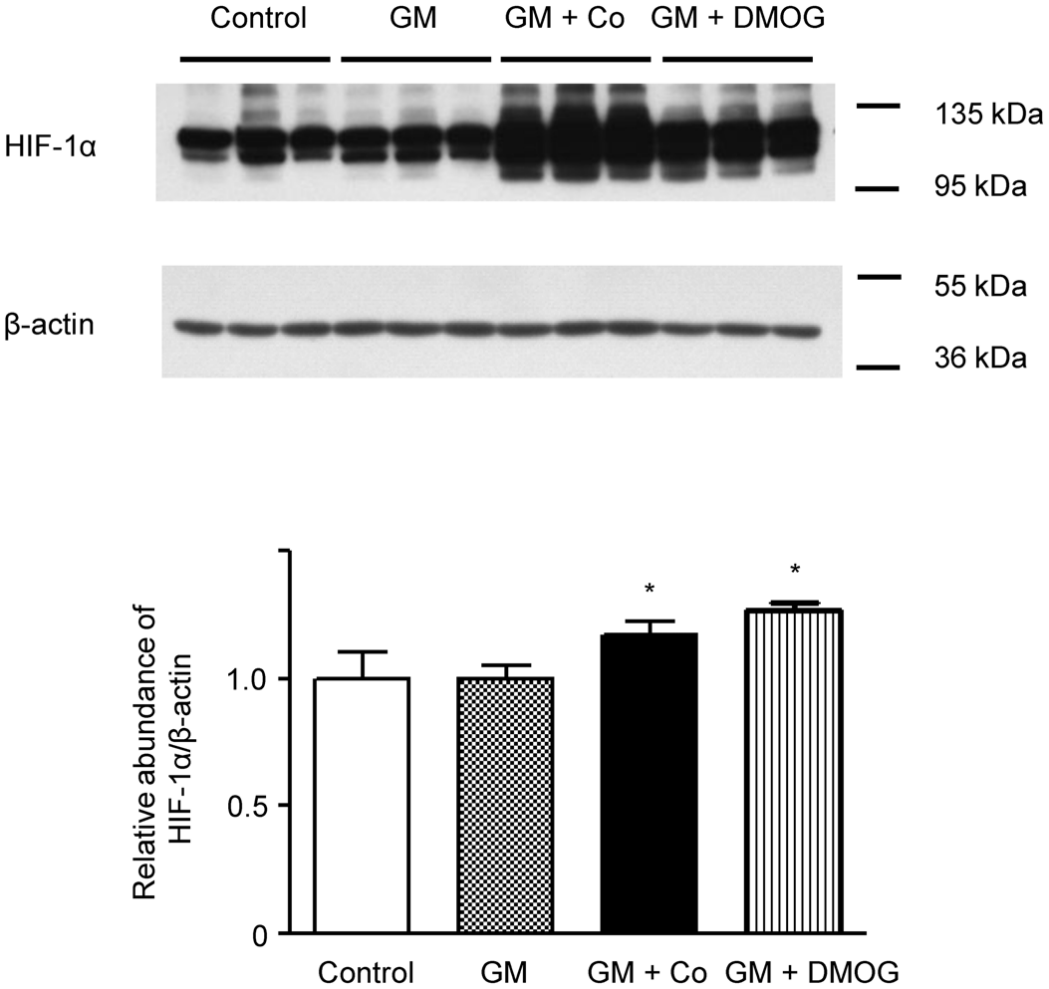
Activation of hypoxia-inducible factor (HIF) in HK-2 cells. Western blotting of HIF-1α in HK-2 cells. HK-2 cells were pre-treated with 150 µM of cobalt or 1 mM of DMOG for 24 h and then treated with 3 mM of gentamicin for another 24 h. Beta-actin was used as a loading control. A representative set of three independent experiments is shown. Densitometric ratios between HIF-1α and β-actin of cells are shown as the relative ratios of experimental groups compared with control cells. The results are expressed as the mean±s.d. *Significantly different with respect to control cells. *P<0.05. GM, gentamicin treatment; GM+Co, cobalt pre-treatment and gentamicin treatment; GM+DMOG, DMOG pre-treatment and gentamicin treatment.

Cobalt and DMOG are PHD inhibitors and can therefore activate HIF. To activate HIF-1α in the human renal proximal tubule cell HK-2, we treated HK-2 cells with cobalt or DMOG. There were no changes in the amount of HIF-1α protein between the control and gentamicin-treated cells (Fig. 1). However, the expression of HIF-1α was significantly increased in the cells pre-treated with cobalt or DMOG.

**Figure 2:**
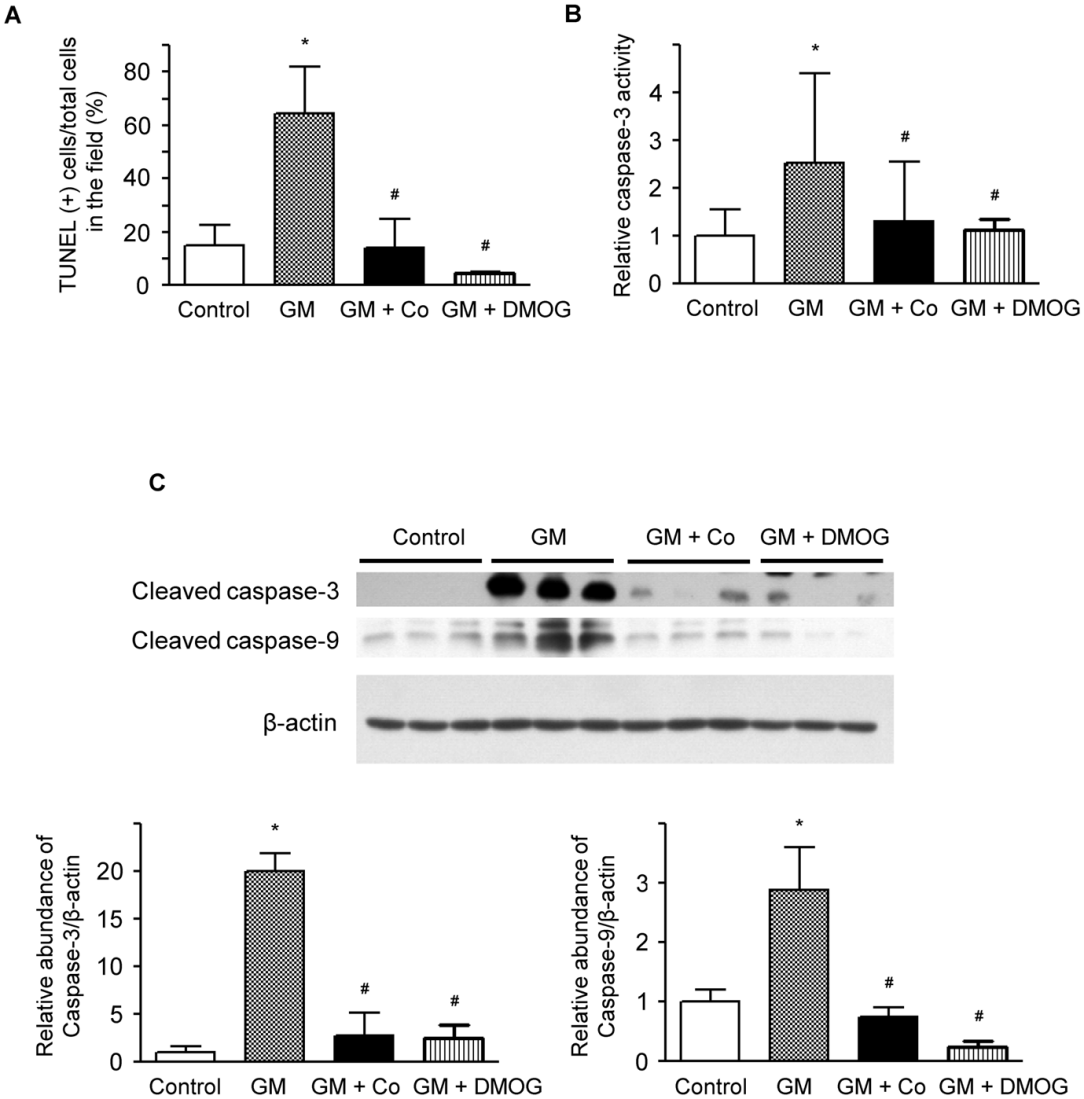
Effects of cobalt or DMOG pre-treatment on gentamicin-induced apoptosis in HK-2 cells. (A) The protective effect of cobalt or DMOG against gentamicin-induced apoptosis in HK-2 cells. The level of apoptosis is presented as the percentage of TUNEL-positive cells among the total cells counted in the field. (B) Effects of cobalt or DMOG on caspase-3 activity in gentamicin-treated HK-2 cells. The cells were harvested, and the activity of caspase-3 was analyzed using ELISA. The caspase-3 activities of the cells are shown as the relative activities of the experimental groups compared with control cells. (C) Effects of cobalt or DMOG on caspase-3 and caspase-9 in gentamicin-treated HK-2 cells. Western blotting was performed with the specific antibody against cleaved caspase-3 and caspase-9. Beta-actin was used as a loading control. Densitometric ratios between caspase-3, 9 and β-actin of cells are shown as the relative ratios of experimental groups compared with control cells. A representative set of three independent experiments is shown. The results are expressed as the mean±s.d. *Significantly different with respect to control cells. ^#^Significantly different with respect to gentamicin-treated cells. *^#^ *P*<0.05. GM, gentamicin treatment; GM+Co, cobalt pre-treatment and gentamicin treatment; GM + DMOG, DMOG pre-treatment and gentamicin treatment.

To determine whether gentamicin-induced apoptosis occurred in HK-2 cells, we treated the cells with gentamicin and analyzed them with enzymatic labeling of DNA strand breaks using terminal deoxynucleotidyl transferase-mediated deoxyuridine triphosphate nick end-labeling (TUNEL). The number of apoptotic cells was markedly increased by treatment with gentamicin (Fig. 2A). We examined the protective effects of HIF activation against apoptosis and found that pre-treatment with cobalt or DMOG significantly reduced gentamicin-induced apoptosis. Caspase-dependent signaling plays a major role in the apoptotic signaling pathway [25]. Caspase-3 activity was markedly elevated by gentamicin treatment (Fig. 2B). The cleaved subtypes of both caspase-3 and caspase-9 were also increased in the cells treated with gentamicin (Fig. 2C). However, pre-treatment with cobalt or DMOG significantly reduced caspase-3 activity and the quantities of cleaved subtypes of both caspase-3 and caspase-9 in gentamicin-treated HK-2 cells. The increase in cleaved caspase-9 was associated with the mitochondria-mediated signaling pathway in apoptosis. These results indicate that HIF activation protects HK-2 cells from gentamicin-induced mitochondria-mediated apoptosis.

**Figure 3:**
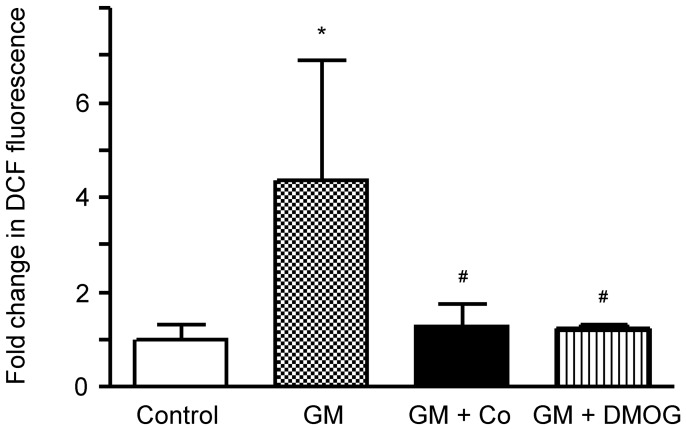
Effects of cobalt or DMOG pre-treatment on gentamicin-induced ROS generation in HK-2 cells. Gentamicin-induced increases in intracellular ROS were revealed by fluorescent intensities of 2′,7′-dichlorofluorescein. The fluorescent intensities of the cells are shown as the relative intensities of the experimental groups compared with control cells. A representative set of three independent experiments is shown. The results are expressed as the mean ± s.d. *Significantly different with respect to control cells. ^#^Significantly different with respect to gentamicin-treated cells. *^#^ *P*<0.05. GM, gentamicin treatment; GM+Co, cobalt pre-treatment and gentamicin treatment; GM+DMOG, DMOG pre-treatment and gentamicin treatment.

Next, we investigated whether HIF activation prevented gentamicin-induced ROS formation because ROS are important mediators of gentamicin-induced apoptosis. The increases in intracellular ROS formation by gentamicin were revealed by fluorescence using 2′,7′-dichlorofluorescein (DCF). As shown in Figure 3, pre-treatment with cobalt or DMOG greatly inhibited gentamicin-induced ROS formation.

**Figure 4:**
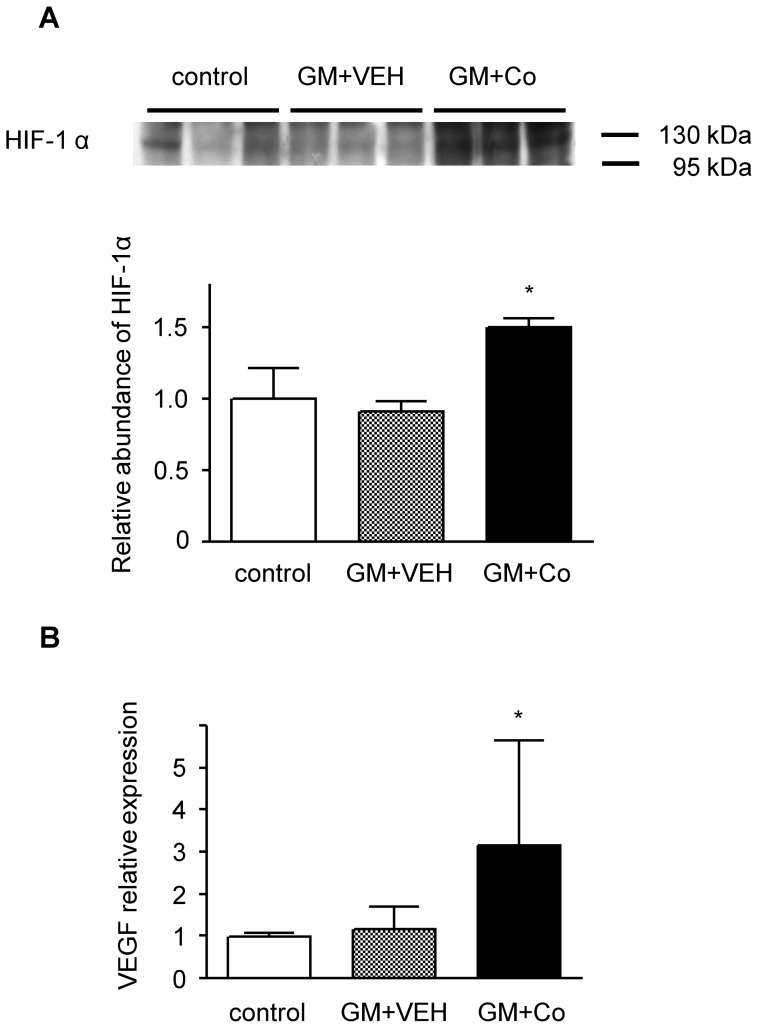
Activation of hypoxia-inducible factor (HIF) in rat kidneys. (A) Representative western blot and group data depicting HIF-1α in the nuclear extracts of kidney homogenates from the experimental animals. The abundance of HIF-1α protein in the control rats was arbitrarily set at 1. (B) VEGF mRNA expression determined by real-time RT-PCR using the total renal RNA of rats from each group. The baseline expression levels of VEGF mRNA in the control rats were arbitrarily set at 1. The results are expressed as the mean±s.d. *Significantly different with respect to control rats and rats treated with gentamicin and vehicle. *P<0.05. GM + VEH, gentamicin and vehicle treatment; GM+Co, gentamicin and cobalt treatment.

We examined whether treatment with cobalt activated HIF-1α *in vivo*. The up-regulation of HIF-1α proteins by cobalt infusion was demonstrated by western blotting of kidney nuclear homogenates. The band density corresponding to HIF-1α was not affected by gentamicin treatment (0.91±0.07-fold vs. the control), but it was significantly increased in the rats treated with cobalt (1.5±0.06-fold vs. the control) (Fig. 4A). Next, we investigated the expression of VEGF to determine whether treatment with cobalt activated the HIF-regulated gene. The level of VEGF mRNA was not affected by gentamicin (1.17±0.53-fold vs. the control), but it was significantly increased by cobalt treatment (3.16±2.49-fold vs. the control) (Fig. 4B).

Table 1 shows the animals’ renal parameters at the end of the experiments. There were no significant differences in the levels of BUN among the three groups of rats. Serum creatinine levels were significantly increased in the gentamicin-treated rats, but they were not affected by cobalt treatment. However, cobalt treatment significantly improved creatinine clearance, which was decreased by gentamicin. Gentamicin treatment also increased proteinuria, but the amount of proteinuria was reduced by half in the rats treated with cobalt. From these results, HIF activation attenuated the renal dysfunction and proteinuria caused by gentamicin.

**Figure 5:**
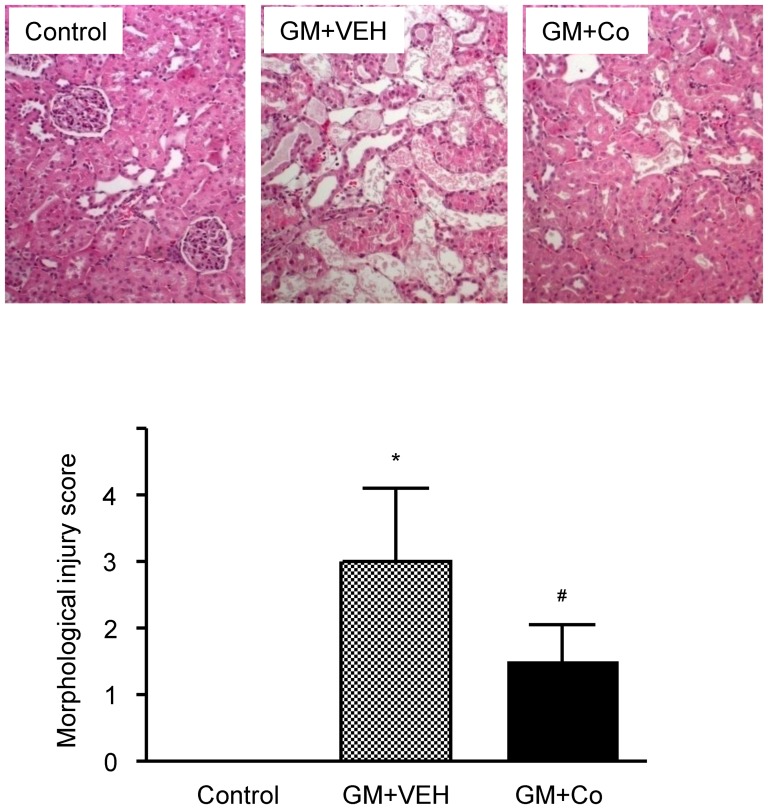
Effects of gentamicin and cobalt on renal histology. Representative pictures of H&E-stained renal cortical areas and morphological injury scores. The results are expressed as the mean ± s.d. *Significantly different with respect to control rats. ^#^Significantly different with respect to the rats treated with gentamicin and vehicle. *^#^ *P*<0.05. GM+VEH, gentamicin and vehicle treatment; GM+Co, gentamicin and cobalt treatment. Magnification×200.

Figure 5 shows a representative histological finding of the kidneys from each group of animals. The degree of renal injury induced by gentamicin was significantly reduced in the rats that received cobalt, compared with the rats that received vehicle (score: 1.5±0.6 vs. 3.0±1.1, cobalt vs. vehicle, *P*<0.05).

**Figure 6:**
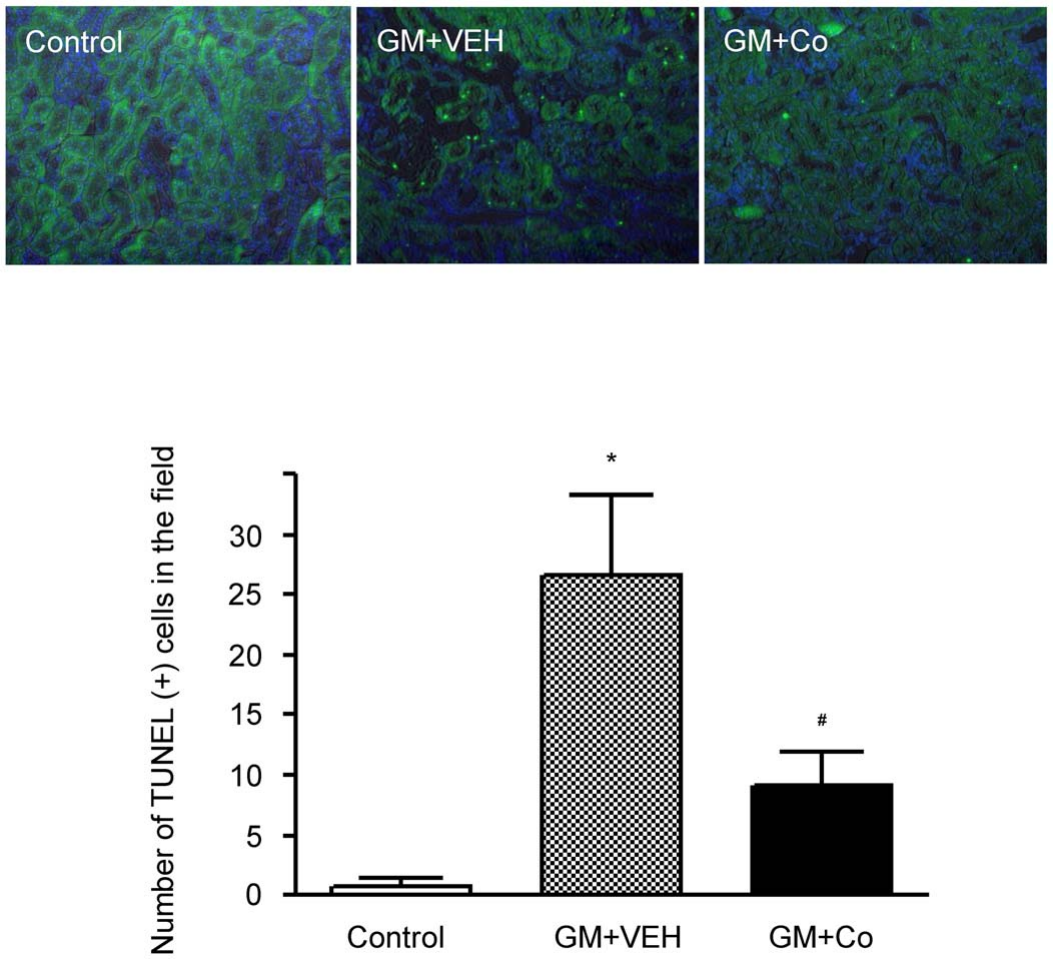
Effects of gentamicin and cobalt on apoptosis in rat kidneys. Apoptotic cells in the kidneys of experimental animals were detected using TUNEL staining. The results are expressed as the mean±s.d. *Significantly different with respect to control rats. ^#^Significantly different with respect to the rats treated with gentamicin and vehicle. *^#^ *P*<0.05. GM+VEH, gentamicin and vehicle treatment; GM+Co, gentamicin and cobalt treatment. Magnification×200.

To analyze gentamicin-induced apoptosis, we examined kidney sections after detecting DNA fragmentations with the *in situ* TUNEL assay. The scattered and bright nuclei stained by the TUNEL assay were easily detected in the kidneys of gentamicin-treated rats, yet the number of nuclei was significantly decreased in the kidneys of the gentamicin-cobalt-treated rats (Fig. 6). This result indicates that the HIF activator cobalt inhibits gentamicin-induced apoptosis in rat kidneys.

**Figure 7:**
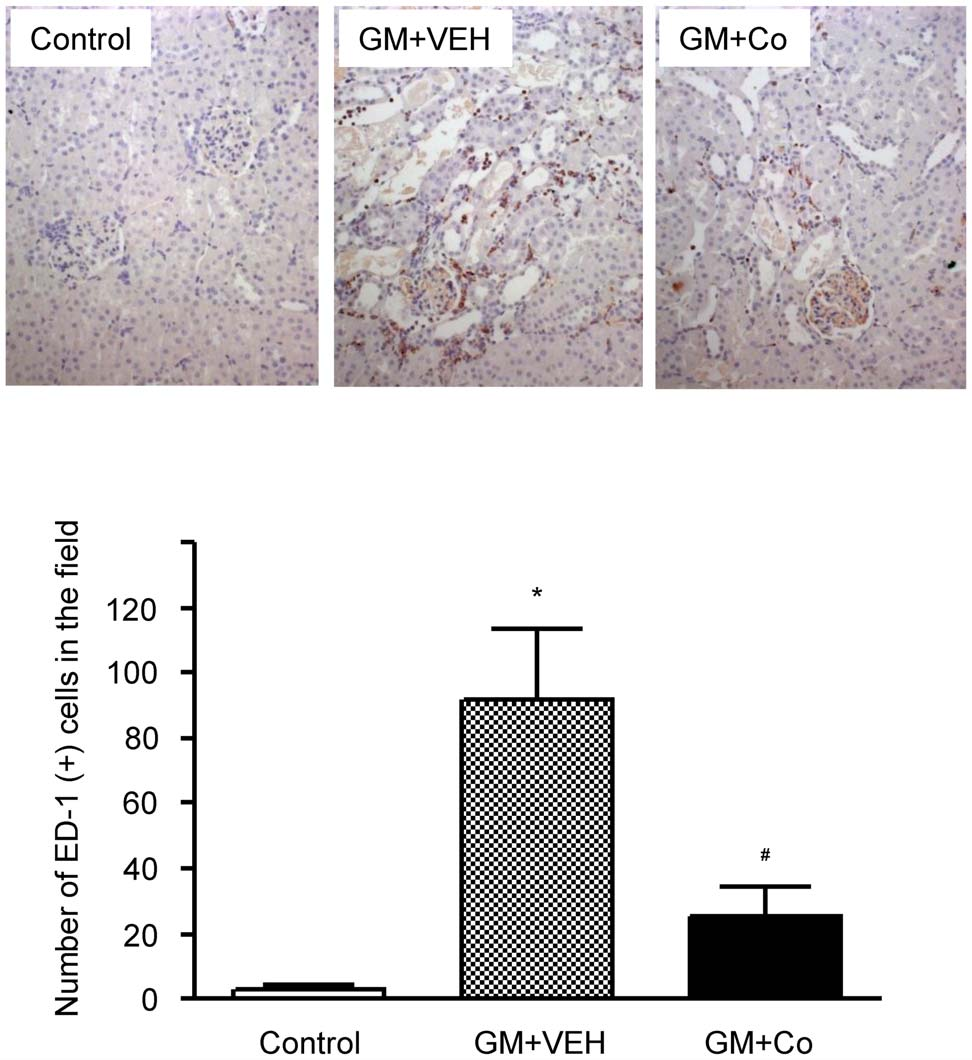
Effects of gentamicin and cobalt on the infiltration of macrophages in rat kidneys. Immunohistochemical stains for ED-1 in representative renal cortices of experimental animals. The number of ED-1-positive cells per field was counted in each group. The results are expressed as the mean±s.d. *Significantly different with respect to control rats. ^#^Significantly different with respect to the rats treated with gentamicin and vehicle. *^#^ *P*<0.05. GM+VEH, gentamicin and vehicle treatment; GM+Co, gentamicin and cobalt treatment. Magnification×200.

Gentamicin treatment induced macrophage infiltration in the tubulointerstitium, as determined by an increase in ED-1-positive cells (Fig. 7). By counting the absolute number of ED-1-positive cells, we observed a marked increase in macrophage infiltration by gentamicin and a significant reduction in response to cobalt treatment. The mean ED-1 counting score was 25.6±8.7 in the cobalt-treated rats and 91.7±22.1 in the vehicle-treated rats.

## Discussion

The main finding of this study is that HIF activation using PHD inhibitors can protect renal tubule cells against gentamicin. This conclusion is based on the consistent results of the *in vitro* and *in vivo* experiments. To the best of our knowledge, this report is the first to show that PHD inhibitors prevent functional, histological kidney injury by gentamicin. These effects are caused by the anti-oxidative, anti-inflammatory, and anti-apoptotic activities of HIF activation.

The injury induced by gentamicin in this study was modest. Acute kidney injury that occurs from gentamicin exposure in clinical practice is rarely severe, with incremental increases in the plasma creatinine that are usually mild (0.5 to 2.0 mg/dL, about 50% decrease of creatinine clearance) [26]. Irreversible kidney damage is uncommon. Animal model in our study is appropriate for gentamicin nephrotoxicity in humans. Therefore, our results could be translated into clinical practice. Animal study also showed that very slight elevation of creatinine or BUN levels were observed in gentamicin-treated rats despite their marked renal histopathological injuries [20]. It is suggested that creatinine or BUN levels are relatively insensitive biomarker for gentamicin nephrotoxicity.

Xu *et al*. showed the elevation of HIF mRNA levels in the rats treated with 3 different doses of gentamicin [20]. It has been reported that gentamicin induces ROS generation [5], and ROS have also been implicated in the stabilization of HIF-1 [14]. However, we did not observe any HIF activation by gentamicin in cells and tissues. Other effects, such as repression of transcription and translation by gentamicin, may override a ROS-mediated HIF stabilization. Differences in the experimental protocols, such as the gentamicin administration routes (intramuscular vs. intraperitoneal) and the methods used for the detection of HIF activation (immunoblotting vs. microarray) may have contributed to these discrepancies.

Oxidative stress plays an important role in the nephrotoxicity of gentamicin [27]. There have been reports that treatment with gentamicin produces oxidative stress in renal tubule cells, both *in vivo* and *in vitro* [28], [29]. Therefore, there have been many attempts to study the effects of antioxidants on the reduction of renal dysfunction and tissue damage induced by gentamicin [6], [27], [30], [31]. However, there has been only one study of the role of antioxidants in modulating the direct cytotoxic effects of gentamicin on renal tubule cells [29]. The antioxidant effect of HIF activation has already been demonstrated in acute and chronic kidney disease models [17], [18], [20]. In this study, ROS was generated by gentamicin, and its generation was reduced by PHD inhibitors, which indicates that HIF activation prevents gentamicin-induced ROS formation in renal tubule cells.

Treatment with gentamicin has been shown to involve renal inflammatory responses in experimental animals [32], [33], [34]. In fact, attempts to protect against gentamicin-induced AKI resulted in an inhibition of the inflammatory responses [33], [35]. In this study, we showed that rats treated with gentamicin displayed a marked increase in macrophage infiltration into the renal tubulointerstitium, but cobalt treatment attenuated the inflammatory response by reducing inflammatory cell accumulation. HIF has a divergent effect on the progression of inflammation depending on in which cell type it is expressed. HIF-1α may be considered pro-inflammatory in that its expression in inflammatory cells of the myeloid lineage contributes to cell survival [36]. In contrast, genetic or pharmacologic activation of HIF was protective in murine experimental colitis [37], [38], [39]. Therefore, HIF activation can be considered to be anti-inflammatory in the intestinal epithelial cells. In a number of models of acute and chronic inflammation, including diseases of the kidney and gastrointestinal tract, recent data have indicated a potential therapeutic role for HIF activation [13], [17], [37], [38], [39].

Gentamicin treatment results in the apoptosis of tubular epithelial cells, both in experimental animals and in cultured cells [40]. Cytosolic gentamicin acts on mitochondria and activates the mitochondrial pathway of apoptosis [41]. In this experiment, gentamicin induced mitochondria-mediated apoptosis *in vitro* and *in vivo*. However, HIF activation using PHD inhibitors protected against this apoptosis. The connection among HIF, inflammation, and apoptosis needs to be elucidated. Cummins *et al*. reported that HIF activation by DMOG reduced the inflammation in a murine model of colitis due to the development of an anti-apoptotic phenotype [39]. Although the anti-apoptotic effect of HIF activation has been reported in cisplatin-induced nephrotoxicity [14], this study was the first to report the anti-apoptotic effect of HIF activation on gentamicin nephrotoxicity.

Our study has some limitations. First, we did not provide additional *in vivo* experimental data using specific PHD inhibitors instead of cobalt. Second, we did not evaluate the effect of direct hypoxic preconditioning on the protection of renal tubule cells against gentamicin. However, carbon monoxide preconditioning and a PHD inhibitor showed comparable levels of HIF activation in an ischemia-reperfusion experiment [13]. Therefore, we can infer from that experiment that hypoxic preconditioning to activate HIF would also show results similar to those from our study. Third, we did not evaluate whether HIF activation affected ROS generation caused by gentamicin in experimental animals.

In summary, the activation of HIF using PHD inhibitors attenuated renal dysfunction, proteinuria, and structural damage in an animal model of gentamicin-induced AKI. This beneficial effect of HIF activation occurred through a reduction of oxidative stress, inflammation, and apoptosis in renal tubular epithelial cells. Currently, HIF PHD inhibitors are in clinical trials for the treatment of anemia [42].Therefore, our results may provide a basis for therapeutic intervention to reduce gentamicin nephrotoxicity using HIF PHD inhibitors. The pharmacological activation of HIF may provide a good therapeutic strategy for gentamicin-induced AKI.
